# Cognitive Efficiency and Fitness-to-Drive along the Lifespan: The Mediation Effect of Visuospatial Transformations

**DOI:** 10.3390/brainsci11081028

**Published:** 2021-08-01

**Authors:** Luigi Tinella, Antonella Lopez, Alessandro Oronzo Caffò, Francesco Nardulli, Ignazio Grattagliano, Andrea Bosco

**Affiliations:** 1 Department of Educational Sciences, Psychology, Communication, University of Bari, 70121 Bari, Italy; antonella.lopez@uniba.it (A.L.); alessandro.caffo@uniba.it (A.O.C.); ignazio.grattagliano@uniba.it (I.G.); andrea.bosco@uniba.it (A.B.); 2 Commissione Medica Locale Patenti Speciali, Azienda Sanitaria Locale-Bari, 70121 Bari, Italy; francesco.nardulli@asl.bari.it

**Keywords:** mental rotation, perspective taking, driving behavior, fitness-to-drive, aging

## Abstract

The way people represent and transform visuospatial information affects everyday activities including driving behavior. Mental rotation and perspective taking have recently been found to predict cognitive prerequisites for fitness-to-drive (FtD). We argue that the relationship between general cognitive status and FtD is mediated by spatial transformation skills. Here, we investigated the performance in the Mental Rotation Test (MRT) and the Perspective-Taking Test (PT) of 175 male active drivers (aged from 18 to 91 years), by administering the Montreal Cognitive Assessment (MoCA) to measure their global cognitive functioning. All participants were submitted to a computerized driving assessment measuring resilience of attention (DT), reaction speed (RS), motor speed (MS), and perceptual speed (ATAVT). Significant results were found for the effect of global cognitive functioning on perceptual speed through the full mediation of both mental rotation and perspective-taking skills. The indirect effect of global cognitive functioning through mental rotation was only found to significantly predict resilience of attention whereas the indirect effect mediated by perspective taking only was found to significantly predict perceptual speed. Finally, the negative effect of age was found on each driving measure. Results presented here, which are limited to male drivers, suggest that general cognitive efficiency is linked to spatial mental transformation skills and, in turn, to driving-related cognitive tasks, contributing to fitness-to-drive in the lifespan.

## 1. Introduction

Spatial reasoning is one of the most important human skills, useful for many daily activities [1]. It involves the mental representations of the shape of objects, their locations, as well as cognitive processes of transformation of objects and movement through space [2]. We think spatially as: (a) behind the wheel, we try to quickly compare the incoming visuospatial information flow with our mental representation of the map we saw earlier, as well as: (b) when preparing a trip, we try to find the best way to arrange luggage in the vehicle’s trunk [1,2]. Often, people seem to be able to encode visuospatial information and adopt it into decisions and complex motor actions that allow them to control high-speed locomotion [3].

Driving requires completing several tasks simultaneously [4]. The risk of crashes is directly influenced by the driver’s cognitive and decision-making abilities [5,6]. The solid relationship between the driver’s cognitive functions and driving performance has been previously established [7,8,9,10], showing how attention, executive functions, visuospatial skills, memory, and psychomotor abilities are essential for safe driving. According to Lappi and Mole [3], driving represents a perfect test-bed to better understand how visuospatial information is processed, especially during high-speed navigation. According to Kunishige et al. [11], both object- and self-based spatial representations are necessary for safe driving. For instance, object-based spatial representation supports the monitoring of the change of both the vehicle’s trajectory and the surrounding environment [11], as when turning at an intersection or driving through a roundabout [12]. Self-based representations, on the other hand, have a key role in planning the route by sensing distances and positional relationships with other vehicles [11,13]. Everyday life activities could require the combination of object- and self-based spatial representations, also requiring the ability to switch between them in a flexible manner (i.e., navigation by car) [11].

Basic components of spatial reasoning are traditionally evaluated by testing spatial mental transformation abilities such as mental rotation and perspective-taking, considered object- and self-based transformations, respectively.

Mental rotation is the ability to imagine the movement of objects and spatial forms [14]. The mental rotation task is assumed to be a spatial transformation task in which respondents are required to encode the representation of an object, and then to rotate that mental representation in order to judge its matching with another object presented in a rotated view [1,15,16]. The processes of encoding (i.e., ascribed to the lateral occipital cortex) and rotation (i.e., motor areas and superior parietal lobe) activate distinct brain areas supporting the execution of the task [1]. Regarding the aforementioned processes, the presence of age differences has been documented in the scientific literature: younger people show better performance in mental rotation than older people [17]. These effects are attributed to age-related decline in spatial working memory tasks [17].

Perspective-taking involves the ability to mentally change the personal perspective by imaging the appearance of an object from a different viewpoint [14]. It is considered a spatial orientation task because it requires one to imagine the transformation of the perceiver’s orientation [14]. In the Object Perspective-Taking Task, respondents are required to imagine standing at the position of one object within the presented configuration, facing another object, and pointing out the direction of a third object (target). Even for this task, the literature suggests age differences: the young outperform older adults [18]. These age-related differences are found because of a reduction in spatial working memory and processing speed during the aging process [18].

Mental rotation and perspective-taking are two highly correlated—but dissociable—imagined transformations [14]. It has been demonstrated that the two spatial abilities are supported by distinct spatial factors [14].

Mental rotation requires imaging manipulation of a perceived object, in an object-to-object frame of reference, in which locations are determined with respect to other objects. Thus, the mental rotation is an object-based (allocentric) manipulation task that relies on spatial visualization processes [19,20]. In an allocentric frame, locations and spatial relations are defined within a framework that is independent of the observer’s viewpoint/location [21,22].

Perspective-taking instead requires imaging oneself in another position, resulting in a self-based (egocentric) spatial transformation (i.e., locations are encoded with respect to the observer’s location) that involves spatial orientation processes [23]. An egocentric frame of reference defines spatial relations and points in the environment relative to the perceiver’s viewpoint/location [21].

Despite mental rotation and perspective-taking relying on separated spatial processes, they are strongly integrated in supporting everyday activities, especially successful navigation by spatial updating during a movement [24,25]. It has been demonstrated that these abilities interact in predicting the accuracy of learned environmental information and they are negatively influenced by using GPS-based systems for navigating. Improvements in these spatial transformation skills were found to be transferable to other visuospatial tasks [26]. In the recent past, it has been demonstrated that mental rotation skills transfer to perspective-taking tests [27,28]. In their studies, authors found that improvements in perspective-taking tasks after mental rotation training were maintained at the follow-up [27,28]. Mental rotation and perspective-taking are both *dynamic* spatial transformations based on rotation skills, but the former is classified as *intrinsic*, i.e., in the object, while the latter is *extrinsic* (i.e., self-based) to the object [26]. The results found by Meneghetti and colleagues [27,28] demonstrated that practicing with object-based rotation tasks may improve performance in a self-based rotation task, positively affecting the ability to imagine different spatial perspectives. Authors provided alternative explanations to these results, suggesting that object-based rotation strategies could have been used to solve self-based transformation tasks (i.e., the object perspective-taking task) [27].

As stated before, mental rotation and perspective-taking skills are involved in the execution of everyday life spatial activities (i.e., staying oriented during walking and driving, or arranging the dishwasher, as well as in video gaming) [1,25]. Although driving activity is often mentioned among the daily activities which require both mental rotation and perspective-taking abilities, evidence of their relationships with objective driving measures is very sparse [29].

Ruginski et al. [30] recently confirmed the association between mental rotation and perspective-taking in supporting spatial tasks. In their study, GPS use was shown to negatively affect environmental learning through its detrimental effect on the two spatial transformation skills. Authors found that perspective-taking fully mediates the effect of mental rotation on environmental learning, demonstrating a mediated–mediation relationship. In other words, mental rotation mediated the negative effects of GPS use on perspective-taking, which, in turn, conveyed this effect on measures of environmental learning. These results provided a conceptual replication of the study of Allen et al. [31] although they were considerably different from those of another similar research study [32] that found that perspective-taking does not mediate the effects of mental rotation on environmental learning outcomes. Thus, GPS-based navigation reduces reliance on the imagined spatial transformation skills, negatively influencing the ability to encode and learn spatial information. It is likely that mental rotation and perspective-taking, by supporting navigation by car, could influence driving behaviors. Despite the importance of object- and self-based spatial representations for safe driving, these assumptions are not verified by investigating together the contribution of both mental rotation and perspective-taking to complex driving maneuvers.

Given these results and considering that navigation through driving combines object- and self-based representations [11,33], it is likely that mental rotation and perspective-taking exert a mediation role between the driver’s cognitive functioning and measures of driving performance.

Recently, we found that both mental rotation and perspective-taking discriminated cognitive fitness-to-drive: mental rotation predicted reaction times, perspective-taking predicted perceptual speed, and both significantly predicted resilience to traffic stress [29].

In the light of the above, it remains unclear how the acquisition, representation, and transformation of spatial information contributes to driving behavior [34]. This sounds surprising given that older drivers, a growing and vulnerable age cohort behind the wheel, are prone to substantial decline in navigation abilities affecting their mobility [11]. For this reason, the present study tries to address this issue from a lifespan perspective.

We aimed to investigate both mental rotation and perspective-taking as mediators of the relationship between overall cognitive functioning and fitness-to-drive, in a sample of male active drivers. According to previous findings [30], perspective-taking fully mediates the effect of mental rotation on spatial learning. The present study replicates and expands this model using cognitive prerequisites for fitness-to-drive as outcomes (i.e., resilience of attention, reaction speed, motor speed, and perceptual speed), the global cognitive functioning as predictor, and both mental rotation and perspective-taking as mediators in a sample of male drivers.

Assessing this model through a series of path analyses, we aimed to compare two hypotheses: (a) mental rotation and perspective-taking independently mediate the relationship between overall functioning and fitness-to-drive; (b) mental rotation and perspective-taking are chained in a mediated mediation, thus explaining the relationship between cognition and fitness-to-drive.

Approaching the literature on the relationship between spatial skills and driving performance, it is unclear whether and how object- and self-based transformation abilities interact in supporting the execution of driving subtasks. The mediation role of mental rotation and perspective-taking on the relationship between overall cognition and driving measures was studied through both single and double mediation in order to investigate both the single contribution and the combined contribution of these spatial skills to complex driving behaviors. Models were tested, controlling for participants’ age as a covariate, in a sample of active male drivers. Considering our previous findings [29] and those presented in the study of Ruginski et al. [30]: (a) the mediated mediation of both the transformation skills was expected to significantly predict measures of both resilience of attention and perceptual speed, (b) mental rotation only is expected to significantly mediate the relationship between cognition and reaction speed, (c) the negative effect of age was expected on each prerequisite for fitness-to-drive [29,35,36,37], and (d) the positive effects of overall cognitive functioning were expected to be present on resilience of attention and perceptual speed but not on reaction and motor speed [29].

## 2. Materials and Methods

### 2.1. Participants

A power analysis to estimate the sample size was carried out using G*Power 3.1 [38], with the following parameters: a p-level of 0.05, a cautious low effect size (0.12), and a power of 0.80. Results indicated that a sample size of 105 participants was adequate to warrant an 80% chance of correctly rejecting the null hypothesis.

One hundred seventy-five healthy active drivers, all males, aged from 18 to 91 years (i.e., age M ± SD = 49.5 ± 21.2; level of education M ± SD = 11.6 ± 3.31, years) were recruited in the study. Participants equally belonged to three different age categories, which are: 61 young (i.e., age range: 18–34 years, age M ± SD = 24.4 ± 4.55), 56 middle-aged (i.e., age range: 35–64 years, age M ± SD = 52.2 ± 7.64), and 58 older adults (i.e., age range: 65–91, age M ± SD = 73.2 ± 6.83). All participants were required to have Italian as their mother tongue and hold a valid current driver’s license, provisional or above; have normal or corrected to normal vision; be in a healthy cognitive state (MoCA inclusion cut-off above 17); have driven at least one time within the last week; and not be or have previously been a professional driver (e.g., taxi driver, truck driver, bus driver, etc.). Descriptive statistics for the whole sample and for the three age groups are reported in Table 1 and in Supplementary Table S1, respectively. The participants, blind to the hypotheses of the study, were volunteers recruited through the support of proxy informants, generally undergraduate students and graduate trainees. All participants signed their informed consent before enrolment in the study.

The Research Ethics Committee of the Department of Education, Psychology, and Communication of the University of Bari approved the study protocol and the whole study was performed following the Helsinki Declaration and its later amendments.

### 2.2. Materials and Procedures

All participants were in a good general state of physical and psychological health. Participants were enrolled between May and September 2019. A brief interview was administered by supervised trainees in clinical neuropsychology to (i) collect demographic information; (ii) exclude neurodegenerative and vision/hearing disorders, and (iii) gather information about participants’ driving rates. After the general anamnesis, eight participants were excluded and did not enter the final sample due to supposed cognitive impairment. After completing the interview, all participants completed the tests described below.

#### 2.2.1. Global Cognitive Functioning

Global cognitive functioning was assessed by using the Montreal Cognitive Assessment (MoCA) [39]. This is a paper-and-pencil cognitive screening which assesses several domains, namely, visuospatial/executive, naming, attention, language, abstraction, memory, and orientation. The raw score is reported on a 30-point scale, adding one point for those participants with <12 years of education. The best cut-off used in an Italian sample is a MoCA score = 17 [40,41] for discriminating participants with probable cognitive impairment.

#### 2.2.2. Measures of Spatial Transformation

The Mental Rotation Test (MRT) [20] was administered to assess participants’ object-based spatial transformation. The MRT is a paper-and-pencil test composed of 20 items (i.e., two-dimensional stimuli representing tri-dimensional figures composed of cubes). Each item includes a criterion figure and four response options (i.e., two correct and two distractors). Correct alternatives have an identical structure to the criterion but are shown rotated. Participants must find the two figures out of four that are rotations of the criterion. The MRT is divided into two parts, and 3 min are given to accomplish each part. The entire procedure takes about 10 min.

The Perspective-Taking Test (PT) [14] was administered to assess participants’ self-based spatial transformation. The PT is a paper-and-pencil test composed of 12 items (i.e., configuration of seven objects drawn on the top half of the sheet paper). Participants were asked to imagine being at the position of one object of the configuration, facing another object, and to indicate the direction to a third object (the target). A circle was drawn in the bottom half of the sheet paper, the imagined standing point was drawn in the center of the circle, and the imagined heading (direction to the second object) was represented by an arrow pointing vertically up. Participants must draw an arrow from the center of the circle pointing to the direction of the target. The item’s score is the absolute directional error, which is the deviation in degrees between the participant’s answer and the correct direction to the target. The total score is the average deviation across items. A high total score corresponds to a lower level of ability in PT. The time limit to accomplish the test is 5 min. The entire procedure takes about 10 min.

#### 2.2.3. Fitness-to-Drive Screening

The Drivesc package of the Vienna Test System [42] was used to assess psychological fitness-to-drive. The Drivesc is a computerized fitness-to-drive evaluation that has already been shown in several studies to have predictive validity in real-world driving performance [43,44]. The apparatus includes an ergonomic response panel, foot pedals, a standard audio output device (headset), and a video screen. The experimental screening takes approximately 25 min and it includes three subtests: Reaction Test (RT; a measure of reaction and motor speed), Determination Test (DT; a measure of resilience of attention) and Adaptive Tachistoscopic Traffic Perception Test (ATAVT; a measure of perceptual speed).

The RT is a simple reaction task that involves the ability to respond to specific auditory and visual stimuli as quickly and accurately as possible. The RT provides two distinct measures, namely reaction speed (i.e., the time between the onset of the stimulus and the moment in which the participant’s finger leaves the rest button) and motor speed (i.e., the time between the moment in which the participant’s finger leaves the rest button and the moment in which the reaction button is pressed). The measures are taped in milliseconds: a short time of reaction (i.e., high visual and motor reaction speed) corresponds to a higher ability to quickly respond.

The DT provides a measure of traffic stress tolerance and resilience of attention. Participants have to react as quickly and accurately as possible to rapidly changing auditory and visual stimuli of different frequency and colors. The software varies the speed of stimuli presentation based on the respondent’s ongoing performance through a computer adaptive system and records participants’ performance in terms of accuracy (i.e., hits, omissions, and false alarms) and response delay (i.e., milliseconds), providing a unique score.

The ATAVT measures the participant’s ability to rapidly identify objects and visual patterns, gaining an overview of the traffic scenario. The ATAVT was administered in the right-hand traffic form according to the Italian traffic laws. The test is composed of pictures of traffic scenarios presented very briefly after an auditory cue. After each picture, the participants must select which one, or more, objects out of five (i.e., motorcycles/bicycles, automobiles, traffic signs, traffic lights, and pedestrians) they have perceived by choosing among listed options. The participants’ performance was the number of correct responses (omissions and false alarms are also recorded).

The scores of the three subtests were provided as a raw score (called parameters) and percentile ranks related to normative data. For the statistical analysis, only percentile ranks have been considered. The entire procedure was made clear to the participants beforehand. Participants were assessed individually in a silent and well-lit room, without disturbances, in the Department of Psychology of the University. Each assessment session was accomplished by two instructed research assistants and lasted 60–90 min, with breaks provided as requested by participants.

### 2.3. Statistical Analysis

Descriptive statistics, preliminary analyses of cognitive tests and measures of fitness-to-drive were performed. To achieve the aims of the present study, a series of path analysis was performed by using the software R, lavaan package [45], testing four mediated–mediation models: the effect of global cognitive functioning through the double mediation of mental rotation and perspective-taking on resilience of attention (model 1), reaction and motor speed (models 2 and 3), and perceptual speed (model 4), respectively, was tested.

**Figure 1:**
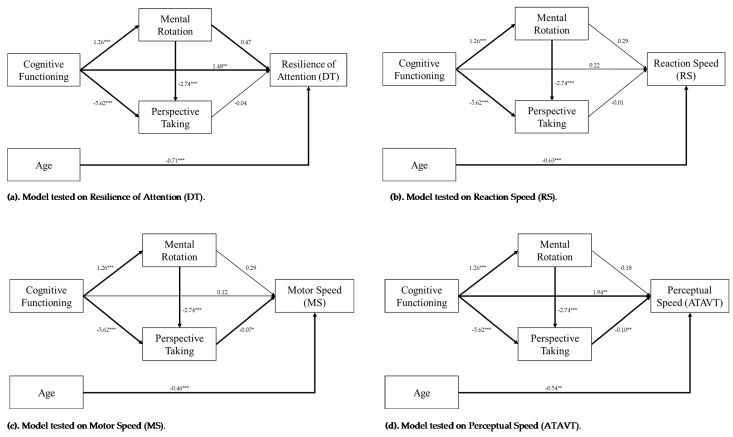
Graphical representations of the four mediated mediation models tested. (**a**) Resilience of Attention, (**b**) Reaction Speed, (**c**) Motor Speed, (**d**) Perceptual speed. Coefficients are reported for each regression path. * indicates *p* < 0.05, ** indicates *p* < 0.01, *** indicates *p* < 0.001. Significant paths (*p* < 0.05) are bolded.

Age, considered as a continuous variable, was controlled as a covariate in all models. Figure 1 shows the path diagrams involving overall cognitive functioning as a predictor, mental rotation and perspective-taking as the two chained mediators, and in turn each of four FtD outcomes. For each outcome, the final mediated––mediation model has been estimated in line with the results of the initial path analysis in which “MoCA -> FtD” outcome association was the primary path, MRT and PT interference paths represented single mediations, and the MRT-PT path represented the double mediation. To assess the goodness of fit of all the models, we used the total R^2^. The 95% CI was used to evaluate the significance of overall and separated indirect effects. The value of *p* was set to 0.05 for the calculation of statistical significance. The effect size of Cohen’s f^2^ was estimated for each model [46].

## 3. Results

Means, standard deviations, and correlation coefficients for the variables employed in the study are shown in Table 1.

### 3.1. Path Analyses 

Prior to performing path analyses, the multicollinearity between mental rotation and perspective-taking was controlled through linear regressions. Despite the variables being strongly correlated (r = −0.541; *p* < 0.001), the variance inflation factor did not exceed 5 (VIF = 1.46) [47]. The effects of MoCA on both mental rotation and perspective-taking have been initially evaluated. Preliminary analysis revealed a significant direct effect of MoCA’s total score on MRT (β = 1.26; *p* < 0.001) and PT (β = −5.62; *p* < 0.001). With respect to the effects of predictors on measures of fitness-to-drive, significant results were found for the negative effect of age on each driving outcome, for the positive effects of MoCA’s total score on DT (β = 1.44, *p* < 0.01) and on ATAVT (β = 1.94; *p* < 0.01), the effect of MRT on DT (β = 0.471, *p* < 0.05), and the effects of PT on both the MS (β = −0.70, *p* < 0.05) and the ATAVT (β = −0.101, *p* < 0.01). Table 2 shows significant and non-significant effects for regression models on each outcome.

#### 3.1.1. Resilience of Attention (DT)

The first mediation analysis was performed, testing the above-mentioned model on measures of resilience of attention (DT). The predictors explained 55% of the variance in DT scores (Table 2). Table 3 shows direct, indirect, and total effects of predictors on DT for the estimated paths. Significant results were found for the direct effect of MoCA (β = 1.44, *p* < 0.01), for the indirect and total effect of MRT (indirect: β = 0.594, *p* < 0.05; total: β = 2.071, *p* < 0.001), and for the total effect of the indirect path mediated by PT (β = 1.731, *p* < 0.01) and by MRT-PT (β = 1.633, *p* < 0.01), but not for their indirect effects (PT: β = 0.254, *p* = 0.148; MRT-PT: β = 0.156, *p* = 0.147). The model showed a large effect size (Cohen’s f^2^ = 1.23).

#### 3.1.2. Reaction Speed (RS)

The second mediation model was performed on measures of reaction speed (RS). The predictors explained 27% of the variance of the RS scores (Table 2). The direct, indirect, and total effects of predictors on RS are reported in Table 3. Non-significant results were found for the direct effect of MoCA (β = 0.221, *p* = 0.718). Regarding the spatial skills’ mediation, both the indirect effects (MRT: β = 0.372, *p* = 0.242; PT: β = 0.080, *p* = 0.721; MRT-PT: β = 0.049, *p* = 0.721) and the total effects (MRT: β = 0.593, *p* = 0.456; PT: β = 0.300, *p* = 0.693; MRT-PT: β = 0.270, *p* = 0.725) resulted in non-significance. The model showed a small effect size (Cohen’s f^2^ = 0.38).

#### 3.1.3. Motor Speed (MS)

The third mediation model was performed on measures of motor speed (MS). Predictors explained 28% of the variance in MS scores (Table 2). Table 3 shows direct, indirect, and total effects of predictors on MS. The direct effect of MoCA was found to be non-significant (β = 0.117, *p* = 0.858) as well as both the indirect path mediated by the MRT (β = 0.363, *p* = 0.170) and its total effect (β = 0.480, *p* = 0.465). Likewise, no significant results were found for the indirect effect of the PT-mediated path (β = 0.392, *p* = 0.056) and the MRT-PT-mediated path (β = 0.241, *p* = 0.055), but not for their own total effects (PT: β = 0.509, *p* = 0.422; MRT-PT: β = 0.358, *p* = 0.572). The model showed a small effect size (Cohen’s f^2^ = 0.39).

#### 3.1.4. Perceptual Speed (ATAVT)

The last mediation model was tested on measures of perceptual speed (ATAVT). The predictors explained 39% of the variance of the ATAVT scores (Table 2). The direct, indirect, and total effects of predictors on ATAVT are reported in Table 3. Significant results were found for the direct effect of MoCA (β = 1.949, *p* < 0.01) and for the total effect of the path mediated by MRT (β = 2.174, *p* < 0.01), but not for the indirect path (β = 0.226, *p* = 0.445). Furthermore, significant results were found for the indirect effects of the PT-mediated path (β = 0.596, *p* < 0.05) and the MRT-PT-mediated path (β = 0.350, *p* < 0.05), as for their total effects (PT: β = 2.518, *p* < 0.05; MRT-PT: β = 2.299, *p* < 0.01). The model showed a moderate effect size (Cohen’s f^2^ = 0.65).

## 4. Discussion

The present study aimed to investigate whether and how the relationship between global cognitive functioning and prerequisites for the fitness-to-drive is mediated by both object- and self-based spatial transformation skills during the lifecycle in a sample of Italian males. Overall, cognitive efficiency positively predicted the execution of computerized driving subtasks in this group, as follows: through the mediation of mental rotation on measures of the resilience of attention (DT), through the mediation of perspective-taking on perceptual speed (ATAVT), as well as through double causal mediation (mental rotation on perspective-taking) on measures of ATAVT.

The way people mentally represent and transform spatial information is a relevant issue for safe driving [34]. Despite their importance in supporting the driving activity, no previous study investigated the mediation role of both mental rotation and perspective-taking in the relationship between cognitive functioning and driving tasks.

As shown, positive and significant relationships were found between cognitive functioning and both spatial (i.e., MRT and PT) and driving measures (i.e., DT, RS, MS, ATAVT). In the same way, both mental rotation and perspective-taking showed significant positive relationships with each driving measure.

First, significant results were found for the positive effects of global functioning on both resilience of attention and perceptual speed in our sample. The higher the MoCA total score, the higher the resilience of attention and perceptual speed. These two driving-related measures tap into stressful driving situations (i.e., driving through traffic) requiring considerable cognitive effort to quickly detect static and dynamic stimuli in the visual field and take rapid decisions. This evidence confirms that the driver’s cognitive functioning constitutes a strong predictive factor of fitness-to-drive [8,9,10,48], also suggesting the usefulness of performing global cognitive screening for driving assessment purposes by using standardized tools [7,29,49,50]. No significant result was found for the effects of the MoCA total score on both reaction and motor speed. This result may have emerged due to different sensorimotor and cognitive components involved in the execution of the Reaction Test and the MoCA, respectively. In line with this, the MoCA showed significant effects on those driving subtasks that involve a larger amount of cognitive resources (i.e., DT and ATAVT) but not on those that involve simple reaction processes (i.e., RS and MS). On the other hand, the MoCA total score also predicted spatial transformation skills (i.e., mental rotation and perspective-taking) positively. This evidence emerged probably because of general cognitive functions that are involved and shared in both the execution of the MoCA subtasks and in the spatial transformation tasks (i.e., visuospatial attention, working memory, abstraction).

Second, results also showed that mental rotation significantly mediated the relationship between global cognitive functioning and resilience of attention in male Italian drivers. Through its positive effect on object-based transformation skills, the driver’s cognitive functioning positively predicted resilience of attention. Previous studies found a positive influence of object-based transformations on driving measures [50,51,52]. For example, performance in the Paper-Folding Test [53] (i.e., an object-based transformation test) was found to be positively associated with safe simulated driving measures [51], and negatively associated with collision rates [54] in young and old drivers. In previous research, we found a causal relationship between mental rotation and resilience of attention, concluding that object-based spatial transformation skills support the safe management of the vehicle, especially in high cognitive demand situations [29]. It is likely that some object-based spatial representation processes (i.e., spatial visualization) involved in solving mental rotations were also required in the execution of the Determination Test (DT; a measure of resilience of attention). Thus, this result probably emerged because of shared object-based spatial cognitive processes between the two employed tasks [1,29]. Moreover, Moen et al. [1] found that training of mental rotation ability improved the participants’ ability to encode spatial relations among the parts of the object, thus leading to more complete spatial mental representations. Indeed, it is plausible that improvements in driving performance could occur as a result of more accurate encoding and more efficient rotation [1]. On the other hand, previous studies demonstrated both that driving simulation exposure had a training effect on mental rotation abilities [55], and the effectiveness of 14-day training of mental rotation abilities in reducing motion sickness [56]. The result presented here extends the previous evidence showing the structural relationships among those variables. Furthermore, this suggested that spatial visualization processes contribute to significantly sustaining resilience of attention to traffic stress in male Italian drivers, with consequences for the accuracy of the vehicle’s management.

Third, the most remarkable observation of this study was obtained through path analysis: mental rotation and perspective-taking fully mediated the effect of cognitive functioning on perceptual speed. In particular, perspective-taking was found to mediate the effect of mental rotation on perceptual speed in a mediated mediation. The result indicates that object-based spatial transformation can have an influence, by supporting the self-based one, on the ability to obtain an overview of the traffic at a glance. It seems that spatial visualization improves the male driver’s visual acquisition of traffic-related information by affecting egocentric perspective-taking. This seems plausible when separately considering the single mediation of both the spatial skills observed in this study. On measures of perceptual speed, the single mediation of perspective-taking, but not that of mental rotation, significantly conveyed the effect of cognitive functioning. These results extend to a complex driving task the evidence shown by Ruginski et al. [30] in which mental rotation and perspective-taking mediated the negative effects of GPS use on environmental learning, through the same mediated–mediation effect presented here.

Overall, the results partially confirmed the hypotheses by showing how mental rotation and perspective-taking are chained in a causal relationship in which the former was found to predict the latter and taken together they mediated the effects of cognitive functioning on perceptual speed. Moreover, the single mediation of spatial transformation skills was found to significantly predict the driving subtasks which largely involved cognitive resources (i.e., reacting under traffic stress conditions and obtaining an overview of the visual scenario) in contrast to those involving simple reaction processes (i.e., reaction and motor speed). This could suggest that cognitive functions affect driving performance by supporting the ability to image spatial transformations of objects and entities of the driving environment (i.e., one’s own vehicle, the other vehicles, the pedestrians, etc.) and the driver’s current viewpoint (i.e., by anticipating one’s own viewpoint before a turn at the intersection, taking the spatial perspective of other drivers during a passing maneuver, etc.) [11,33].

Moreover, the relationship between visuospatial skills and driving performance seems to show positive mutual effects in which the former improves the latter and vice versa [55,56]. Previous research demonstrated a positive relationship between simulated driving and visuospatial abilities in older drivers [57]. Improvements in spatial cognition were observed even in people with different degrees of cognitive impairment and dementia after the repetitive use of driving simulation [48]. On the other hand, some studies demonstrated the effectiveness of visuospatial abilities training in reducing symptoms of motion sickness during simulated driving [48,55]. These recent studies demonstrated that visuospatial abilities could be trained through exposure to a challenging and dynamic visuospatial environment [55]. Considering the results of the present study and those of previous research [56], it seems possible to conclude that both mental rotation and perspective-taking could be considered key measures that subserve the assessment of cognitive fitness-to-drive and the training of impaired driving capabilities [29,56].

Finally, age significantly and negatively affected all the driving subtasks. In agreement with previous studies [35,49,50], this result suggests that with the increase of age, the male driver’s driving abilities decrease due to age-related decline in cognitive and psychomotor functioning [58,59,60,61,62], with consequences for driving capabilities. The negative correlation between age and cognition observed in this study supported this conclusion.

The present study presented some limitations that could be overcome through theoretical and methodological implementations. First, we used a convenience sample of male drivers. Gender comparisons reflecting gender-related differences in spatial transformation skills could reveal a different contribution of spatial skills on FtD in men and women. Further studies could clarify if these models could find clinical applicability even on a population of female drivers. Moreover, future research may need to replicate the models on a sample representative of the general population in order to clarify whether and how these results can be applied to non-Italian drivers and professional drivers. Second, the use of a unique cut-off score of MoCA in screening for cognitive impairment in young participants, adults, and older participants may lead to biased results. Future investigations need to overcome this limitation with appropriate cut-off scores for young and adult drivers. Finally, the employment of more ecological measures of driving performance with respect to those used in this study (i.e., simulated driving and real-world driving) may help to clarify whether the effects of both mental rotation and perspective-taking would be observable even on simulated and/or real-world driving [63]. Similarly, models presented here could benefit from the inclusion of measures of topographical representations (i.e., egocentric and allocentric) [22,64,65] in order to also study the role of large-scale spatial representations, thus making possible a more complex predictive model.

## 5. Conclusions

Mobility largely contributes to human well-being, particularly in elders [66]. Driving a vehicle represents the most common way to travel [67] not only because it means independence and a convenient form of transport but also because maintaining driving ability symbolizes autonomy and competence [48,67]. With the increase of age, decline of visuospatial cognitive functioning affects mobility, making the drivers prone to driving cessation [11,67]. Results from the present study showed the specific role of spatial transformation skills in that relationship.

In conclusion, the complex interaction between cognitive functions in supporting driving activity is best expressed by the combination of object- and self-based spatial transformations skills during the driving activity. Protocols for both the assessment and the training of fitness-to-drive could benefit from the present results by considering the evaluation of spatial transformation skills as measured by useful standardized tasks able to both predict [29] and train [56] the driver’s fitness to drive during the lifecycle. Psychologists, practitioners, and occupational therapists of mobility centers could find the employment of spatial transformation measures useful in both cognitive assessment and training/recovery of driving abilities for licensing, relicensing, and license renewal at all ages.

## Figures and Tables

**Table 1:** Means, standard deviations, and correlations between variables used in path analysis. MoCA: Montreal Cognitive Assessment, MRT: Mental Rotation Test, PT: Perspective-Taking, DT: Determination Test, RS: Reaction Speed, MS: Motor Speed; ATAVT: Adaptive Tachistoscopic Traffic Perception Test. All correlations are significant, *p* < 0.001.

	Mean	SD	AGE	MoCA	MRT	PT	DT	RS	MS	ATAVT
AGE	49.5	21.2	—							
MoCA	24.2	2.93	−0.445	—						
MRT	16.4	9.13	−0.478	0.405	—					
PT	89.6	58.7	0.427	−0.454	−0.541	—				
DT	52.1	28.3	−0.717	0.496	0.521	−0.474	—			
RS	45.6	29.2	−0.529	0.278	0.338	−0.286	0.540	—		
MS	48.2	24.5	−0.523	0.309	0.392	−0.399	0.454	0.416	—	
ATAVT	40.5	30.3	−0.573	0.468	0.418	−0.473	0.554	0.362	0.408	—

**Table 2:** Path analysis results: standardized estimates, standard errors, Z scores, *p* value, and *R* squared (explained variance) for each outcome and each mediation variable.

Effect	Estimate	Standard Error	Z	*p*	*R* Squared
*On Mental Rotation (MRT)*					0.164
of MoCA	1.259	0.215	5.857	<0.001	
*On Perspective-Taking (PT)*					0.359
of MoCA	−5.626	1.325	−4.264	<0.001	
of MRT	−2.748	0.426	−6.450	<0.001	
*On Resilience of Attention (DT)*					0.552
of Age	−0.712	0.072	−9.837	<0.001	
of MoCA	1.447	0.587	2.514	<0.01	
of MRT	0.471	0.184	2.566	<0.05	
of PT	−0.045	0.029	−1.539	0.124	
*On Reaction Speed (RS)*					0.267
of Age	−0.635	0.098	−6.506	<0.001	
of MoCA	0.221	0.792	0.278	0.781	
of MRT	0.296	0.248	1.194	0.233	
of PT	−0.014	0.040	−0.359	0.720	
*On Motor Speed (MS)*					0.280
of Age	−0.456	0.080	−5.669	<0.001	
of MoCA	0.117	0.652	0.719	0.858	
of MRT	0.288	0.204	1.412	0.158	
of PT	−0.070	0.033	−2.140	<0.05	
*On Perceptual Speed (ATAVT)*					0.394
of Age	−0.543	0.092	−5.920	<0.01	
of MoCA	1.949	0.744	2.619	<0.01	
of MRT	0.179	0.233	0.770	0.441	
of PT	−0.101	0.037	−2.728	<0.01	

**Table 3:** Direct, indirect, and total effects of MoCA on resilience of attention (DT), reaction speed (RS), motor speed (MS), and perceptual speed (ATAVT). * indicates *p* < 0.05, ** indicates *p* < 0.01.

Effect	Direct Effect	Indirect Effect MRT	Total Effect MRT	Indirect Effect PT	Total Effect PT	Indirect Effect MRT-PT	Total Effect MRT-PT
On DT	1.478 **	0.594 *	2.071 **	0.254	1.731 **	0.156	1.633 **
On RS	0.221	0.372	0.593	0.080	0.300	0.049	0.270
On MS	0.117	0.363	0.480	0.392	0.509	0.241	0.358
On ATAVT	1.949 *	0.226	2.174 **	0.569 *	2.518 *	0.350 *	2.299 **

## Supplementary Materials

The following are available online at https://www.mdpi.com/article/10.3390/brainsci11081028/s1, Table S1: Means and Standard Deviations of variables used in path analysis for the three age groups. MoCA Montreal Cognitive Assessment, MRT Mental Rotation Test, PT Perspective Taking, DT Determination Test, RS Reaction Speed, MS Motor Speed; ATAVT Adaptive Tachistoscopic Traffic Perception Test.
