# Supplementary material for: Cognitive Efficiency and Fitness-to-Drive along the Lifespan: The Mediation Effect of Visuospatial Transformations
Source: Brain Sci. 2021 Aug 1;11(8):1028. doi: 10.3390/brainsci11081028 (PMC8392112; doi:10.3390/brainsci11081028)
Supplement: Supplementary file 1 [file brainsci-11-01028-s001.zip › brainsci-1213502-supplementary.pdf]

	Young (N = 61)	Middle-aged (N = 56)	Older adults (N = 58)
Age, years	24.4 ± 4.55	52.2 ± 7.64	73.2 ± 6.72
Education, years	12.1 ± 2.44	11.3 ± 3.69	11.5 ± 3.68
MoCA	25.7 ± 2.68	24.5 ± 2.72	22.5 ± 2.50
MRT	21.9 ± 9.85	15.7 ± 8.16	11.4 ± 4.72
PT	60.2 ± 50.2	90.2 ± 57.7	120 ± 52.9
DT	75.2 ± 19.4	53.6 ± 21.8	26.4 ± 18.3
RS	62 ± 26.4	48.3 ± 27	25.7 ± 21.5
MS	63.5 ± 20.1	45.9 ± 21.2	34.3 ± 23
ATAVT	59.3 ± 27.8	43 ± 27	18.3 ± 20.2

Table S1: Means and Standard Deviations of variables used in path analysis for the three age groups. MoCA Montreal Cognitive Assessment, MRT Mental Rotation Test, PT Perspective Taking, DT Determination Test, RS Reaction Speed, MS Motor Speed; ATAVT Adaptive Tachistoscopic Traffic Perception Test.
